# Supplementary material for: Prevalence of Undernutrition and Effect of Body Weight Loss on Survival among Pediatric Cancer Patients in Northeastern Hungary
Source: Int J Environ Res Public Health. 2021 Feb 4;18(4):1478. doi: 10.3390/ijerph18041478 (PMC7914605; doi:10.3390/ijerph18041478)
Supplement: Supplementary file 1 [file ijerph-18-01478-s001.zip › Supplementary Table S1.docx]

|  |  | OVERALL SURVIVAL | | | EVENT FREE SURVIVAL | | |
| --- | --- | --- | --- | --- | --- | --- | --- |
|  |  | HR | CI | p | HR | CI | p |
| At diagnosis | BW total  H  S | 3.36  4.01  2.53 | 1.47-7.71  1.05-15.32  0.85-7.52 | 0.004  0.42  0.093 | 2.94  4.15  2.16 | 1.29-6.68  1.09-15.81  0.73-6.34 | 0.01  0.037  0.16 |
|  | WFH total  H  S | 2.25  0.94  3.72 | 0.89-5.65  0.12-7.13  0.27-10.85 | 0.084  0.95  0.016 | 1.96  0.94  3.12 | 0.78-4.92  0.12-7.02  1.08-9.02 | 0.14  0.95  0.035 |
|  | BMI total  H  S | 3.03  1.39  4.54 | 1.17-7.85  0.17-11.21  1.48-13.97 | 0.022  0.75  0.008 | 2.67  1.46  3.87 | 1.03-6.87  0.18-11.8  1.28-11.71 | 0.04  0.71  0.016 |
|  | IBW% total  H  S | 1.82  0.89  2.71 | 1.1-3.02  0.35-2.224  1.45-5.07 | 0.019  0.8  0.002 | 1.62  0.8  2.56 | 1.0-2.63  0.32-1.99  1.39-4.71 | 0.05  0.63  0.003 |
| During therapy | BW total  H  S | 3.46  7.2  1.59 | 1.87-6.41  2.59-20.48  0.68-3.72 | 0.00  0.00  0.28 | 2.9  7.65  1.22 | 1.59-5.29  2.75-21.3  0.53-2.81 | 0.001  0.00  0.63 |
|  | WFH total  H  S | 3.09  2.85  2.67 | 1.76-5.43  1.1-7.38  1.3-5.49 | 0.00  0.03  0.00 | 2.82  2.81  2.29 | 1.64-4.83  1.1-7.17  1.16-4.51 | 0.00  0.03  0.016 |
|  | BMI total  H  S | 3.91  5.98  2.47 | 2.18-7.00  2.13-16.74  1.19-5.14 | 0.00  0.00  0.015 | 3.63  6.16  2.16 | 2.08-6.33  2.22-17.03  1.09-4.3 | 0.00  0.00  0.027 |
|  | IBW% total  H  S | 0.84  1.85  3.79 | 0.7-1.0  0.79-4.31  1.25-8.24 | 0.063  0.15  0.00 | 1.86  1.5  2.4 | 1.12-3.08  0.68-3.28  1.22-4.71 | 0.015  0.31  0.01 |
|  | WL% > 10% total  H  S | 2.49  2.95  1.98 | 1.46.4.24  1.25-6.29  0.97-4.04 | 0.001  0.13  0.06 | 1.92  2.39  1.43 | 1.17-3.15  1.07-5.31  0.74-2.78 | 0.00  0.032  0.28 |
|  | WL% 20-30% total  H  S | 4.13  6.79  2.15 | 2.1-8.12  2.37-19.47  0.82-5.58 | 0.0  0.194  0.11 | 3.08  5.34  1.49 | 1.62-5.87  1.97-14.44  0.59-3.72 | 0.001  0.001  0.39 |
| End of treatment | BW total  BW H  BW S | 2.69  1.0  1.7 | 0.77-7.54  0.46-6.2 | 0.117  0.41 | 3.92  1.0  3.65 | 1.31-11.78  0.65-6.28 | 0.015  0.22 |
|  | WFH total  WFH H  WFH S | 1.67  1.0  1.66 | 0.37-7.54  0.3-8.24 | 0.5  0.53 | 5.12  1.0  3.65 | 1.68-15.58  1.14-11.7 | 0.004  0.029 |
|  | BMI total  BMI H  BMI S | 2.93  1.0  2.38 | 0.38-22.26  0.3-18.68 | 0.29  0.4 | 13.19  1.0  8.4 | 2.93-59.33  1.77-40.52 | 0.001  0.007 |
|  | IBW% total  IBW% H  IBW% S | 0.78  1.02  1.78 | 0.6-1.02  0.12-8.21  0.75-4.34 | 0.08  0.97  0.2 | 2.4  0.89  1.66 | 1.21-4.78  0.11-7.04  0.74-3.68 | 0.012  0.91  0.21 |

Supplementary Table S1.: Five-years overall and event-free survival rate in all patients and subgroups in three time periods

BW: body weight, WFH: weight-for-height, BMI: body mass index, IBW%: ideal body weight percent, WL%, weight loss percent

H: hematological mailgnancy, S: solid tumor

HR: Hazard Ratio, CI: confidention interval, p: level of significance
