# Supplementary figures and images for: Prevalence of Undernutrition and Effect of Body Weight Loss on Survival among Pediatric Cancer Patients in Northeastern Hungary
Source: Int J Environ Res Public Health. 2021 Feb 4;18(4):1478. doi: 10.3390/ijerph18041478 (PMC7914605; doi:10.3390/ijerph18041478)

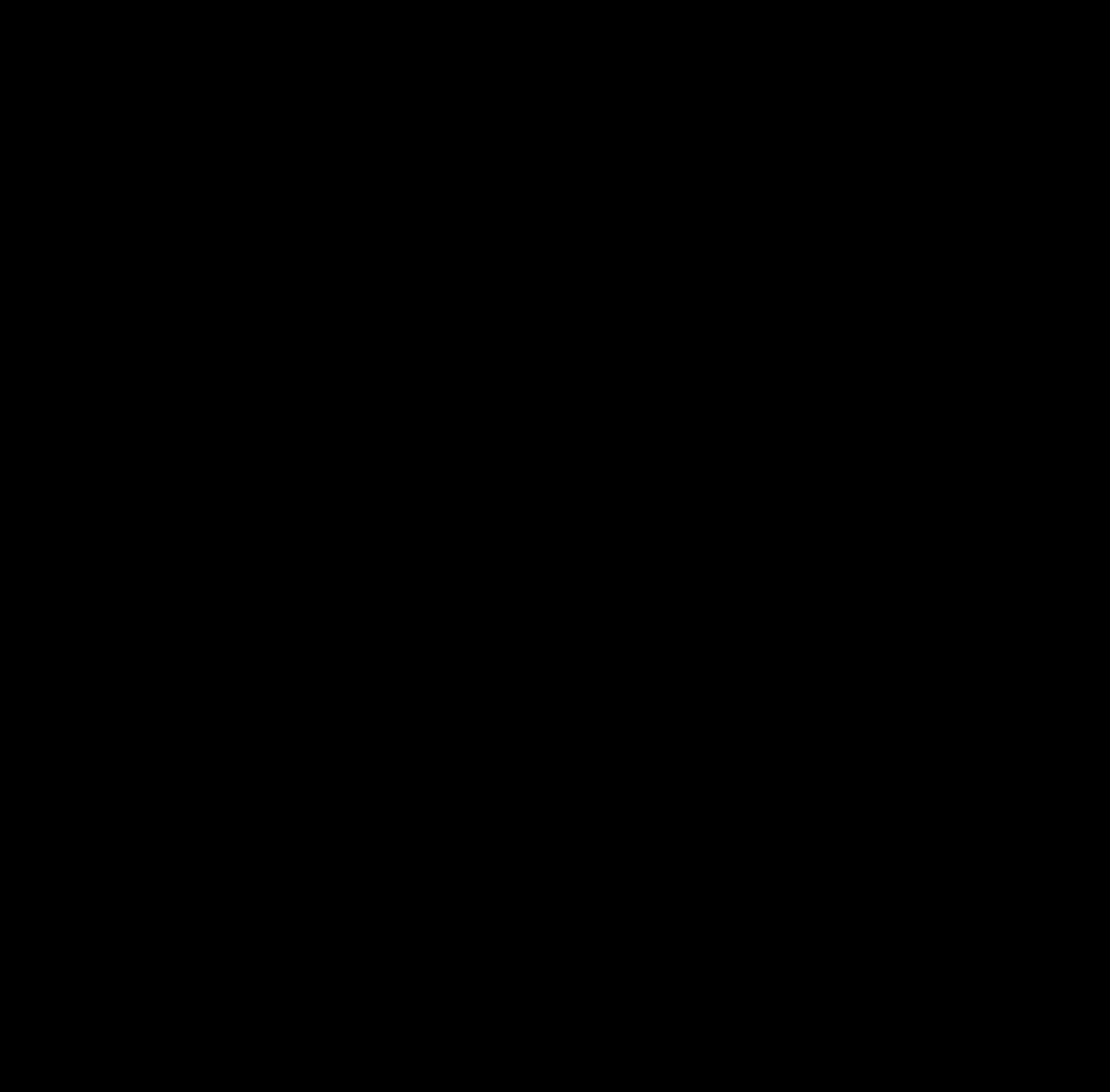

Supplement: Supplementary file 1 [file ijerph-18-01478-s001.zip › Supplementary Figure S1..tif]
